# Infantile tibia vara: Treatment of Langenskiold stage IV

**DOI:** 10.4103/0019-5413.41861

**Published:** 2008

**Authors:** Salil P Umrani, Alaric J Aroojis

**Affiliations:** Department of Pediatric Orthopedics, Bai Jerbai Wadia Hospital for Children, Parel, Mumbai - 400 012, India

**Keywords:** Blount's disease, hemiplateau elevation, infantile tibia vara

## Abstract

An eight year old girl presented with a progressively increasing deformity of the left proximal tibia since last 2 years. She had no history of trauma, fever and swelling of left knee. There were no obvious signs of rickets/muscular dystrophy. She had 25 degrees of tibia vara clinically with lateral thrust and a prominent fibular head. The radiograph of left knee revealed tibia vara with medial beaking and a significant depression of the medial tibial epiphysis and metaphysis. A computed tomography (CT) scan revealed significant depression of the medial tibial epiphysis but no bony bar in the physis or fusion of the medical tibial epiphysis. There was a posterior slope in addition to the medial one. She was treated with elevation of the medial tibial hemiplateau with subtuberosity valgus derotation dome osteotomy. She also underwent a lateral proximal tibial hemiphysiodesis (temporary stapling). A prophylactic subcutaneons anterolateral compartment fasciotomy was also performed. All osteotomies united in 2 months. All deformities were corrected and she regained a knee range of 0 to 130 degrees. At final followup (4 years), there was no recurrence of varus deformity, knee was stable, with 1cm of leg length discrepancy. In Langenskiold stage IV tibia vara, elevation of medial tibial plateau, a subtuberosity valgus derotation osteotomy and a concomitant lateral hemiephiphysiodesis has given good results.

## INTRODUCTION

Infantile tibia vara, or Blount's disease, is a developmental disorder of growth that affects the medial aspect of the proximal tibial epiphysis, physis and metaphysis leading to progressive varus deformity of the knee in young children.^1^–^3^ This leads to deviation of the mechanical axis and malorientation of the joint resulting in poor function, instability and degenerative changes. Blount's disease is rare in the Indian subcontinent and hence other etiologies of tibia vara like trauma, infection and rickets need to be ruled out.

## CASE HISTORY

**Figure 1:**
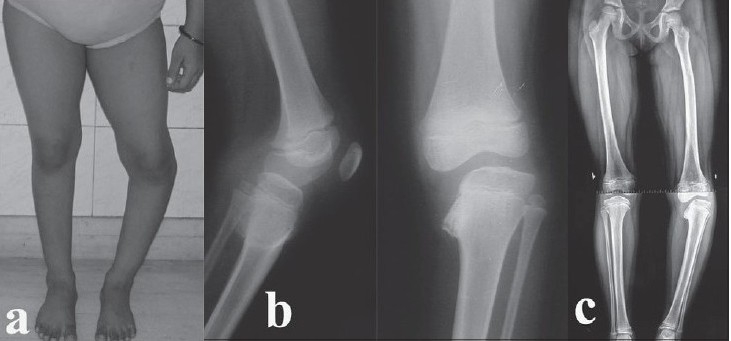
(a) Preoperative clinical photograph of an eight-year-old girl showing a varus deformity of the left proximal tibia. (b) Preoperative lateral and anteroposterior radiographs showing medial beaking and a significant depression of the medial tibial epiphysis and metaphysis (Langenskiold Stage IV). (c) Preoperative alignment-view radiograph of both lower limbs showing a medial mechanical axis deviation and demonstrating the extent of the deformity

An eight-year-old girl presented with a progressively increasing varus deformity of the left proximal tibia since two years [Figure 1a]. She had no history of trauma or fever and swelling of left knee previously. Functionally, she had pain during exercises and had difficulty in running and participating in sports activities, besides cosmetic concerns. She was of average build with no obvious signs of rickets. There was no muscle atrophy and the knee range of motion was 0-130°. The knee was stable in full extension, but there was a distinct laxity on the medial aspect in 20° of flexion (the Siffert-Katz sign)^4^ suggesting a posteromedial defect. The tibial varus was about 25° (measured clinically with a hand-held goniometer), with a lateral thrust and a prominent fibular head. The tibial intorsion was about 20° as evidenced by the thigh-foot angle. There was an apparent shortening of about one cm of left leg due to the deformity. There were no patellofemoral signs or symptoms. The biochemical parameters were within normal limits.

Standard antero-posterior and lateral radiographs of the left knee demonstrated tibia vara with medial beaking and a significant depression of the medial tibial epiphysis and metaphysis [Figure 1b]. The mechanical axis deviation was assessed on a standing full-length radiograph of both lower limbs with the patellae facing forward (alignment view) [Figure 1c], which showed: (i) Mechanical axis femoro-tibial angle of 20° of varus. (ii) Lateral distal femoral angle of 80° (Normal 88° ± 3°). (iii) Femoral condyle and tibial shaft angle of 60°. (iv) Angle of depression of medial tibial plateau of 58°.

**Figure 2:**
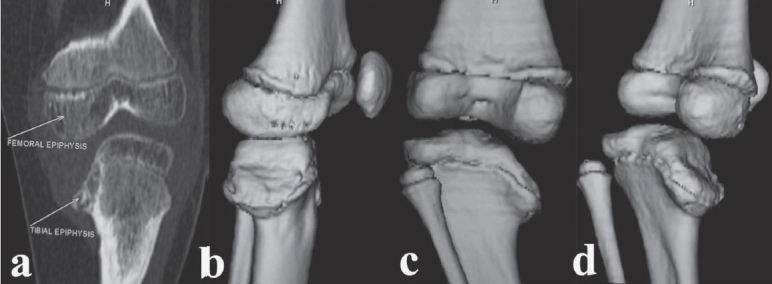
Preoperative CT scan (a) (coronal reformatted image) showing the extent of depression of the medial tibial plateau. Preoperative CT scan (b,c,d) (3D reconstruction) in various rotations demonstrating the posteromedial tibial plateau depression

A computed tomography (CT) scan demonstrated a significant depression of the medial tibial epiphysis but no bony bar in the physis or fusion of the medial tibial epiphysis (Stage IV) [Figure 2a]. The 3D reconstruction showed that there was a posterior slope in addition to the medial one already seen on the plain radiographs measuring about 63° [Figure 2 b,c,d].

**Figure 3:**
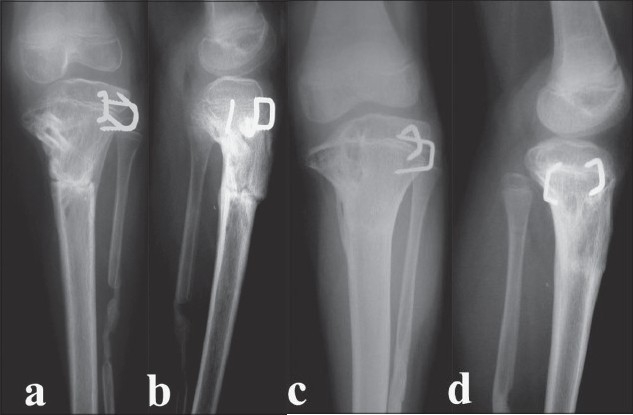
Anteroposterior and lateral (a,b) radiographs taken two months postoperatively. The osteotomies have united, the medial tibial plateau has been satisfactorily elevated and the cortical strut grafts are consolidating well. Anteroposterior and lateral (c,d) radiographs taken two years postoperatively showing maintenance of correction and excellent alignment. There is no recovery of growth in the medial tibial physis

A correction of the intra-articular deformity as well as restoration of the mechanical alignment was planned. The intraoperative arthrogram showed a hypertrophied medial meniscus and cartilage and also demonstrated the true orientation of the knee joint-line. She was treated with elevation of the medial tibial hemiplateau with a subtuberosity valgus derotation dome osteotomy at the apex of the center of rotation of angulation (CORA) (which was calculated on the radiographs). The distal femoral valgus being less than 10° was left alone. In view of the questionable growth potential of the medial proximal tibial physis, she also underwent a lateral proximal tibial hemiepiphysiodesis (temporary stapling) in order to achieve a horizontal knee joint-line, a normal valgus alignment and to prevent subsequent recurrence of the deformity. All these osteotomies were done through a standard anterior-midline approach. The medial elevating osteotomy was supported with cortical strut grafts from the ipsilateral fibula and fixed with a 2-mm Kirschner wire. The subtuberosity valgus derotation osteotomy was fixed with two crossed 2.4-mm Steinmann pins and incorporated in an above-knee plaster while the lateral hemiepiphysis was stapled. A prophylactic subcutaneous anterolateral compartment fasciotomy was also performed. The plaster was changed at intervals till all osteotomies were united (two months) and subsequently the knee was mobilized [Figure 3a,b]. All the deformities were corrected and she regained a knee range of motion from 0-130°.

**Figure 4:**
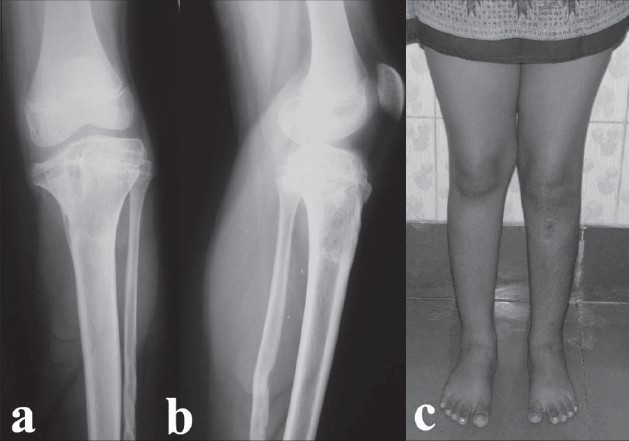
Radiographs (a,b) at final follow-up after percutaneous proximal tibial and fibular epiphysiodesis. Clinical photograph (c) at final (four-year) follow-up showing full correction of deformity and excellent alignment

She was then followed up clinically and radiologically at six-monthly intervals to look for recurrence of varus or overcorrection due to resumption of growth on the medial side. On follow-up, the status of the medial tibial physis was studied closely and it was found that even at two years of follow-up there did not appear to be any recovery in the growth on the medial side [Figure 3c,d]. Hence, a decision was made to proceed with a formal percutaneous epiphysiodesis of the proximal tibial and fibular growth plates [Figure 4a,b]. At final follow-up (after four years), the deformity is well corrected and the knee range of movement remains the same at 0-130° [Figure 4c]. The knee is stable. She has a leg length discrepancy of one cm and it is estimated that she may need a contralateral epiphysiodesis or an ipsilateral limb lengthening at the end of skeletal maturity.

## DISCUSSION

The typical radiographic features of infantile tibia vara in the antero-posterior view are a varus angulation at the epiphyseal-metaphyseal junction; widened and irregular medial physeal line; medially sloped, irregularly ossified, sometimes triangular epiphysis; prominent beaking of the medial metaphysis, with lucent cartilage islands within the beak; and lateral subluxation of the proximal tibia.^1^,^4^,^5^ Langenskiold has described a six-stage radiographic classification of infantile tibia vara that is based on changes observed as the child matured.^5^ Our patient was in Stage IV as there was a depression of the medial tibial plateau, but there was no demonstrable physeal bar or break in the epiphysis.

Radiologically, the standard antero-posterior radiograph shows an oblique view of the proximal tibia, resulting in underestimation of the slope of medial tibial epiphysis. To understand the complex geometry of the proximal tibial epiphysis, CT and magnetic resonance imaging (MRI) have been shown to be useful in assessing the epiphyseal plate to determine the presence of a physeal bony bridge.^6^,^7^ Arthrography defines the true joint-line, which is not always obvious on plain radiographs.^8^ Also, an arthrogram with varus-valgus stress views may be more useful in showing instability; it being a dynamic study. Three-dimensional CT scans are much easier to interpret and are particularly useful in identifying a posterior slope or a central depression which cannot be readily seen on plain radiographs.

The accepted treatment of Blount's disease in the older child (age more than five years) with advanced (Langenskiold Stages IV, V and VI) involvement includes elevation of the medial proximal tibial hemiplateau; a subtuberosity valgus tibial osteotomy with derotation and a lateral proximal tibial epiphysiodesis.^3^,^9^–^12^ This reduces the potential for recurrent varus deformity since the child is still growing. Only valgisation has a high risk of recurrence and is no longer acceptable.

Medial joint-line depression is controversial in infantile tibia vara. Siffert and Katz^4^ described the finding of hypertrophied medial meniscus in conjunction with depression in the posterior half of the proximal tibial articular surface. Other workers^3^,^9^–^13^ have confirmed these findings and have advocated elevation of the medial tibial hemiplateau. In contrast, Stanitski, Stanitski and Trumble^7^ have correlated data from plain radiographs, MRI and arthrogram (in operative cases) and have found that there is no depression of the medial tibial joint-line early on in Blount's disease owing to the hypertrophied medial meniscus and the relatively larger cartilaginous portion of the epiphysis as compared to its ossified portion. Langenskiold and Riska^5^ noted the arthrogram findings of one of their Stage IV patients and found that the joint-line depression was not as much as that of the medial proximal tibial plateau. We believe that there definitely exists a depression in the posteromedial part of the epiphysis, but it is not as severe as the radiograph indicates. Hence elevation of the medial proximal tibial plateau should be done only as much as is necessary to restore a horizontal knee joint-line. We feel that a combination of preoperative 3D-CT scan along with an intraoperative arthrogram is one of the best imaging tools to simultaneously delineate the complex geometry of the proximal tibia in advanced Blount's disease and to define the extent of the posteromedial joint-line depression.

Once the medial tibial plateau is elevated and supported by a wedge graft, the knee becomes stable, thus allowing a valgus derotation osteotomy to be performed at a subtuberosity level as determined by the center of rotation of angulation (CORA).^10^ However, this osteotomy must be placed distal to the tibial tubercle to prevent damage to the tibial apophysis and subsequent genu-recurvatum.^3^ A valgus osteotomy can be done in the tibial shaft by removing a laterally based wedge of bone,^10^ but we believe this would lead to further shortening of an already shortened limb. A dome osteotomy^14^,^15^ safely permits correction of varus, intorsion and procurvatum with very little alteration of limb length. A concomitant osteotomy of the fibula is necessary to permit adequate correction of the genu varum and internal tibial torsion.^3^ The fibular osteotomy also provides struts for elevating the medial tibial hemiplateau^9^ thereby obviating any need for another incision for an iliac crest graft.

The role of lateral epiphysiodesis is also controversial as there is fear of significant shortening at skeletal maturity. However, recent studies^9^,^10^,^12^ have clearly demonstrated the importance of lateral epiphysiodesis to prevent recurrence of varus deformity with growth. Stage IV (Langenskiold) presents a unique entity in which there is no demonstrable physeal bar or fusion but the status of the proximal tibial physis is questionable as regards to potential for further growth.^2^,^3^ Total physeal closure at the time of osteotomy prevents recurrence but by producing unacceptable shortening in young patients, predictably commits them to subsequent limb-lengthening procedures.^2^ Stapling the lateral aspect of the tibial physis inhibits its growth and thereby allows more time for the medial aspect of the physis to recuperate from previous years of abnormal compression.^3^ If the tibia starts to demonstrate increased valgus angulation, the staples can be removed to allow equivalent growth from both sides of the physis.^3^

## CONCLUSION

We report a case of unilateral infantile Blount's disease in Stage IV Langenskiold with four years follow-up treated successfully by elevation of the medial tibial plateau a subtuberosity valgus derotation osteotomy and a concomitant lateral hemiepiphysiodesis.
